# Public Awareness of Invasive Fungal Diseases — United States, 2019

**DOI:** 10.15585/mmwr.mm6938a2

**Published:** 2020-09-25

**Authors:** Kaitlin Benedict, Noelle Angelique M. Molinari, Brendan R. Jackson

**Affiliations:** 1 Division of Foodborne, Waterborne, and Environmental Diseases, National Center for Emerging and Zoonotic Infectious Diseases, CDC.

Fungal diseases range from minor skin and mucous membrane infections to life-threatening disseminated disease. The estimated yearly direct health care costs of fungal diseases exceed $7.2 billion (*1*). These diseases are likely widely underdiagnosed (*1*,*2*), and improved recognition among health care providers and members of the public is essential to reduce delays in diagnoses and treatment. However, information about public awareness of fungal diseases is limited. To guide public health educational efforts, a nationally representative online survey was conducted to assess whether participants had ever heard of six invasive fungal diseases. Awareness was low and varied by disease, from 4.1% for blastomycosis to 24.6% for candidiasis. More than two thirds (68.9%) of respondents had never heard of any of the diseases. Female sex, higher education, and increased number of prescription medications were associated with awareness. These findings can serve as a baseline to compare with future surveys; they also indicate that continued strategies to increase public awareness about fungal diseases are needed.

Porter Novelli’s Fall 2019 ConsumerStyles survey was used to ask, “Have you ever heard of the following infections?” Possible answers were aspergillosis, *Candida* infection or candidiasis, coccidioidomycosis (“Valley fever”), *Cryptococcus* infection, blastomycosis, histoplasmosis, or none of these. The online survey was sent to a nationally representative sample of 4,677 participants aged ≥18 years who were part of the market research and consulting firm Ipsos’ KnowledgePanel. Panel members were randomly recruited by mail using address-based probability-based sampling and were provided with a laptop or tablet computer and Internet access if needed; 3,624 completed the survey, for a response rate of 77.5%. Data were weighted to adjust for sampling design and nonresponse to be representative of the U.S. adult population based on the Consumer Population Survey benchmarks for sex, age, race/ethnicity, education, U.S. Census region, household income, home ownership status, metropolitan area status, and Internet access.

Descriptive and bivariate analyses were used to identify potential factors associated with awareness using simple weighted logistic regression of awareness of each fungal disease by various sociodemographic and health care utilization characteristics. Multivariate weighted logistic regression was estimated to derive adjusted odds ratios (AORs); p-values <0.05 were considered statistically significant. Measures of goodness of fit assessed included Akaike Information Criterion, Max-scaled R-squared, and McFadden’s pseudo R-squared. All analyses were conducted using SAS survey procedures (version 9.4; SAS Institute). No personally identifying information was included in the data file provided to CDC.*

Fewer than one third of participants (31.1%) had ever heard of any of the fungal diseases listed on the survey. Awareness was lowest for blastomycosis (4.1%), followed by aspergillosis (5.1%), histoplasmosis (7.5%), coccidioidomycosis (7.6%), cryptococcosis (9.0%), and candidiasis (24.6%) (Table 1). Persons aware of one fungal disease were more likely to be aware of others (i.e., awareness was correlated among diseases, p<0.001). Female sex, higher educational level, and increased number of prescription medications were associated with awareness in the multivariable models for all 6 fungal diseases (Table 2). Specifically, females were more than three times as likely to be aware of candidiasis (AOR = 3.40, 95% CI = 2.8–4.1, p<0. 001) compared with males. Each additional prescription medication was associated with 6%–11% increased odds of awareness (all p<0.01). Non-White or multiracial respondents had lower odds of awareness of candidiasis (AOR = 0.68, p<0.001) and coccidioidomycosis (AOR = 0.60, p<0.05) compared with White respondents. Residence in the West was associated with significantly higher odds of coccidioidomycosis awareness (AOR = 2.87, p<0.001) compared with residence in the Midwest. Likelihood of blastomycosis awareness was lower for respondents in the Northeast (AOR = 0.52, 95%CI = 0.29–0.91) and the South (AOR = 0.44, 95% CI = 0.27–0.72) than in the Midwest.

**TABLE 1:** Characteristics of respondents who reported ever having heard of certain fungal infections — Porter Novelli Fall ConsumerStyles Survey, United States, 2019

Characteristics	Weighted no. (%)						
	Full sample	Aspergillosis	*Candida* infection or candidiasis	Coccidioidomycosis (Valley fever)	*Cryptococcus* infection	Blastomycosis	Histoplasmosis
**Total**	3,624 (100)	184 (5.1)	881 (24.6)	273 (7.6)	321 (9.0)	148 (4.1)	268 (7.5)
**Sex**							
Male	1,756 (48.4)	69 (4.0)^†^	251 (14.4)*	114 (6.5)^†^	137 (7.9)^§^	66 (3.8)	107 (6.2)^†^
Female	1,868 (51.6)	115 (6.2)	631 (34.1)	159 (8.6)	184 (10)	82 (4.5)	161 (8.7)
**Age group (yrs)**							
18–29	771 (21.3)	26 (3.4)	119 (15.8)*	44 (5.8)^†^	56 (7.4)	21 (2.8)	36 (4.8)*
30–44	894 (24.7)	44 (5.0)	202 (22.9)*	56 (6.3)^†^	80 (9.0)	38 (4.3)	52 (5.9)*
45–59	906 (25.0)	51 (5.7)	244 (27.1)*	71 (7.8)^†^	93 (10.3)	44 (4.9)	71 (7.9)*
≥60	1,052 (29.0)	63 (6.0)	316 (30.2)*	103 (9.8)^†^	93 (8.9)	45 (4.3)	109 (10.4)*
**Race**							
White	2,815 (77.7)	148 (5.3)	720 (25.9)^†^	226 (8.1)^§^	253 (9.1)	119 (4.3)	223 (8.0)^§^
Non-White or multiracial	809 (22.3)	36 (4.5)	162 (20.2)^†^	47 (5.8)^§^	68 (8.5)	30 (3.7)	45 (5.7)^§^
**Ethnicity**							
Non-Hispanic	3038 (83.8)	162 (5.4)	739 (24.6)	226 (7.5)	273 (9.1)	134 (4.5)^§^	240 (8.0)^†^
Hispanic	586 (16.2)	22 (3.8)	142 (24.3)	47 (8.0)	48 (8.2)	14 (2.4)^§^	28 (4.8)^†^
**Education**							
Less than high school	390 (10.7)	11 (2.9)*	48 (12.4)*	28 (7.4)^†^	13 (3.4)*	11 (2.9)^†^	21 (5.6)*
High school	1,038 (28.7)	28 (2.8)*	199 (19.5)*	46 (4.5)^†^	56 (5.5)*	22 (2.1)^†^	46 (4.5)*
Some college	1,024 (28.3)	51 (5.0)*	268 (26.4)*	84 (8.2)^†^	102 (10.0)*	44 (4.3)^†^	76 (7.5)*
Bachelor's degree or higher	1,172 (32.3)	94 (8.0)*	367 (31.6)*	115 (9.9)^†^	150 (12.9)*	72 (6.2)^†^	124 (10.7)*
**Have a child aged <18 years**	982 (27.1)	38 (3.9)^§^	248 (25.6)	67 (6.9)	90 (9.3)	40 (4.1)	58 (6.0)^§^
**MSA category**							
Nonmetropolitan	491 (13.6)	18 (3.7)	104 (21.4)	24 (5.0)^†^	28 (5.8)^†^	21 (4.3)	38 (7.9)
Metropolitan	3133 (86.4)	166 (5.4)	778 (25.1)	249 (8.0)^†^	293 (9.5)^†^	128 (4.1)	230 (7.4)
**Census region^¶^**							
Northeast	643 (17.7)	34 (5.3)	170 (26.9)	29 (4.5)*	46 (7.3)	23 (3.6)*	33 (5.1)^†^
Midwest	755 (20.8)	39 (5.2)	187 (25.0)	52 (7.0)*	69 (9.2)	54 (7.2)*	78 (10.4)^†^
South	1,367 (37.7)	67 (4.9)	320 (23.6)	56 (4.2)*	132 (9.7)	37 (2.7)*	103 (7.6)^†^
West	860 (23.7)	44 (5.2)	205 (24.2)	136 (16.0)*	75 (8.8)	34 (4.1)*	55 (6.5)^†^
**Characteristic**	**Mean (range)**	**Mean (standard error)**					
**Age in years**	47 (18–94)	55 (1.0)^§^	56 (0.5)*	57 (0.9)^†^	54 (0.8)	54 (1.2)	57 (0.9)^†^
**No. of health care provider visits in the last 12 mos**	5 (0–368)	6 (0.6)	6 (0.2)	6 (0.5)	6 (0.4)	5 (0.6)	6 (0.5)
**No. of prescription medications**	2 (0–36)	3 (0.2)^†^	3 (0.1)*	3 (0.2)^†^	3 (0.2)^†^	3 (0.3)^†^	3 (0.2)*
Household income**	$72,106 (<$5,000 to ≥$250.000)	$83,971 ($3,972)^†^	$77,999 ($2,449)*	$76,925 ($3,137)^†^	$78,699 ($2,960)^†^	$80,397 ($4,621)^†^	$82,051 ($2,457)^†^

**Abbreviation:** MSA = metropolitan statistical area.

*p<0.001.

^†^p<0.005.

^§^p<0.10 for F-test. P value for the F-test for significant effect of variable overall, equivalent to t-test for dichotomous and continuous variables.

^¶^ *Northeast*: Connecticut, Maine, Massachusetts, New Hampshire, New Jersey, New York, Pennsylvania, Rhode Island, Vermont; *Midwest*: Illinois, Indiana, Iowa, Kansas, Michigan, Minnesota, Missouri, Nebraska, North Dakota, Ohio, South Dakota, Wisconsin; *South*: Alabama, Arkansas, Delaware, District of Columbia, Florida, Georgia, Kentucky, Louisiana, Maryland, Mississippi, North Carolina, Oklahoma, South Carolina, Tennessee, Texas, Virginia, West Virginia; *West*: Alaska, Arizona, California, Colorado, Hawaii, Idaho, Montana, Nevada, New Mexico, Oregon, Utah, Washington, Wyoming.

** Household income means and standard errors were mapped from the following income categories: 1 = <$5,000; 2 = $5,000 to $7,499; 3 = $7,500 to $9,999; 4 = $10,000 to $12,499; 5 = $12,500 to $14,999; 6 = $15,000 to $19,999; 7 = $20,000 to $24,999; 8 = $25,000 to $29,999; 9 = $30,000 to $34,999; 10 = $35,000 to $39,999; 11 = $40,000 to $49,999; 12 = $50,000 to $59,999; 13 = $60,000 to $74,999; 14 = $75,000 to $84,999; 15 = $85,000 to $99,999; 16 = $100,000 to $124,999; 17 = $125,000 to $149,999; 18 = $150,000 to $174,999; 19 = $175,000 to $199,999; 20 = $200,000 to $249,999; 21 = ≥$250,000.

**TABLE 2:** Adjusted odds ratios (AORs) for sociodemographic and health care utilization characteristics associated with awareness of fungal diseases — Porter Novelli Fall ConsumerStyles Survey, United States, 2019

Characteristic	AOR (95% CI)					
	Aspergillosis	*Candida* infection or candidiasis	Coccidioidomycosis (Valley fever)	*Cryptococcus* infection	Blastomycosis AOR	Histoplasmosis AOR
**Sex (female versus male)**	1.67 (1.18–2.38)^†^	3.40 (2.8–4.13)*	1.53 (1.15–2.04)^†^	1.34 (1.02–1.75)^†^	1.15 (0.78–1.70)	1.51 (1.13–2.02)^†^
**Age, yrs**	1.00 (0.99–1.01)	1.01 (1.01–1.02)*	1.01 (1.00–1.02)	1.00 (0.99–1.01)	1.00 (0.99–1.01)	1.01 (1.00–1.02)
**Race (non-White or multiracial versus White)**	0.75 (0.45–1.25)	0.68 (0.52–0.89)^†^	0.60 (0.39–0.91)^†^	0.84 (0.58–1.21)	0.74 (0.41–1.33)	0.63 (0.39–1.01)^§^
**Ethnicity (Hispanic versus non-Hispanic)**	1.04 (0.60–1.82)	1.38 (1.01–1.87)^†^	0.89 (0.55–1.43)	1.20 (0.77–1.89)	0.65 (0.31–1.37)	0.78 (0.47–1.32)
**Education (referent = bachelor’s degree or higher)**						
Less than high school	0.33 (0.13–0.82)^†^	0.23 (0.13–0.41)*	0.62 (0.30–1.30)	0.22 (0.10–0.47)*	0.52 (0.20–1.34)	0.59 (0.29–1.17)
High school	0.31 (0.18–0.54)*	0.40 (0.3–0.52)*	0.40 (0.25–0.63)*	0.37 (0.25–0.54)*	0.31 (0.17–0.56)*	0.39 (0.26–0.59)*
Some college	0.48 (0.32–0.73)^†^	0.65 (0.52–0.82)*	0.73 (0.53–1.02)	0.67 (0.48–0.92)^†^	0.64 (0.42–0.98)^†^	0.64 (0.45–0.91)^†^
**Have child aged <18 yrs**	0.78 (0.49–1.23)	1.21 (0.95–1.56)	1.04 (0.70–1.54)	1.06 (0.76–1.48)	0.94 (0.59–1.52)	0.85 (0.57–1.28)
**No. of health care provider visits in the last 12 months**	0.99 (0.97–1.01)	1 (0.99–1.00)	0.99 (0.98–1.01)	1 (0.99–1.00)	0.99 (0.98–1.01)	1.00 (0.99–1.01)
**No. of prescription medications**	1.11 (1.03–1.19)^†^	1.06 (1.03–1.10)^†^	1.08 (1.02–1.15)^†^	1.11 (1.05–1.16)*	1.09 (1.02–1.16)^†^	1.10 (1.04–1.16)^†^
**Household income** ^¶^	1.01 (0.96–1.06)	0.99 (0.97–1.02)	1.00 (0.96–1.04)	1.00 (0.97–1.04)	1.01 (0.96–1.07)	1.03 (1–1.07) ^§^
**MSA category (non-MSA versus MSA)**	0.80 (0.45–1.40)	0.90 (0.68–1.19)	0.74 (0.46–1.20)	0.69 (0.45–1.06)^§^	1.17 (0.72–1.91)	1.13 (0.78–1.65)
Census region**						
Northeast	1.17 (0.67–2.04)	1.17 (0.87–1.56)	0.67 (0.40–1.13)	0.87 (0.56–1.35)	0.52 (0.29–0.91)^†^	0.51 (0.34–0.79)^†^
Midwest	Ref	Ref	Ref	Ref	Ref	Ref
South	1.07 (0.65–1.78)	0.99 (0.77–1.28)	0.58 (0.36–0.93)^†^	1.15 (0.79–1.67)	0.44 (0.27–0.72)^†^	0.79 (0.56–1.13)
West	1.10 (0.65–1.88)	1.03 (0.78–1.36)	2.87 (1.94–4.25)*	1.02 (0.68–1.53)	0.69 (0.42–1.12)	0.72 (0.47–1.10)
**Max-rescaled R squared**	0.06	0.15	0.12	0.06	0.06	0.07

**Abbreviations:** CI = confidence interval; MSA = metropolitan statistical area; Ref = referent.

*p<0.001.

^†^p<0.05.

^§^p<0.10.

^¶^Household income included 21 categories, ranging from <$5,000 to >$250,000 yearly. AOR is therefore interpreted as the effect on odds of awareness associated with an increase in income from the mean category ($60,000–$74,999), to the next highest category ($75,000–$84,999).

## Discussion

Public awareness of fungal diseases is low, a concerning finding because these diseases are associated with substantial illness, death, and economic cost, although their true burden remains largely unquantified (*1*,*2*). Primary prevention of fungal diseases can be challenging, particularly for those acquired via inhalation from the natural environment. Therefore, awareness is critical to help prevent severe disease, because early diagnosis and treatment can prevent incorrect treatment and improve outcomes. For example, knowledge of coccidioidomycosis before seeking health care has been associated with faster diagnosis (*3*).

Previous analyses show that coccidioidomycosis awareness is high in Arizona (97%) (*4*) and lower in California (42%) (California Department of Public Health, unpublished data, 2020). More than 95% of cases occur in these two states, with most cases concentrated in Arizona’s Sonoran Desert and California’s southern San Joaquin Valley (*5*). The results suggest much lower levels of awareness in the West (of which Arizona and California account for 87% of the population) than previous studies (*4*,*5*), possibly because of methodologic differences. The lower awareness of coccidioidomycosis among non-White and multiracial respondents is noteworthy given that Black race and Filipino ethnicity are risk factors for severe or disseminated disease. Focused messaging could be useful for these groups (*6*).

Public awareness of histoplasmosis and blastomycosis has not been studied, although public health surveillance shows that 15% of patients with histoplasmosis reported awareness of the disease before their diagnosis, and many were aware because they worked in the health care field (*7*). In 2018, the United States had approximately 5.3 million health diagnosing and treating practitioners nationwide (1.6% of the U.S. population). Some survey respondents might have been familiar with fungal diseases through their occupations; this is supported by the correlation between awareness of each disease. Low blastomycosis awareness is consistent with the disease being considerably less common than coccidioidomycosis and histoplasmosis. Notably, regional awareness patterns for blastomycosis and coccidioidomycosis corresponded to geographic areas where they are more prevalent. Nonetheless, more widespread awareness is essential because these geographic areas appear to be wider than previously appreciated (*8*), and travel-associated cases occur regularly.

Candidiasis can include severe bloodstream infections and other invasive disease, as well as skin and mucous membrane infections. Higher awareness about candidiasis compared with other diseases in this analysis is not surprising given that vulvovaginal *Candida* infections are common, resulting in nearly 1.4 million outpatient visits per year nationwide (*1*). Nonetheless, the fact that only one third of women had heard of candidiasis contrasts with the commonly cited (but not well documented) estimate that 75% of women have at least one vaginal *Candida* infection during their lifetime (*9*), suggesting that many women might know this condition by another name. Future studies might examine familiarity with the more common term “yeast infection.”

In general, mold appears to be well recognized as a potential health risk; for example, 96% of residents surveyed in a posthurricane setting answered “yes” to “do you think mold can make people sick?” (*10*). However, no previous evaluations of awareness about nonallergic health conditions from mold, specifically invasive mold infections, the most common of which is aspergillosis, could be found. Although an estimated 15,000 U.S. hospitalizations occur annually with aspergillosis (*1*), typically involving severe illness, the disease might not be widely known because invasive aspergillosis most commonly affects severely immunocompromised persons. This is supported by the finding of the association between awareness and increasing number of prescription medications, a proxy for health status.

The findings in this report are subject to at least four limitations. First, the data were self-reported. The extent, accuracy, and source of participants’ knowledge could not be determined. Second, no information was available about participants’ health literacy, although greater awareness at higher educational levels was apparent. Third, as shown by the goodness of fit measures, a great deal of the variation in awareness of these fungal diseases remains to be explained, and some might be idiosyncratic based on experiences of family or friends with these diseases. Finally, information about risk factors such as immunosuppression, environmental exposures, and occupation was not available but could help public health and health care professionals develop targeted prevention messages to groups at high risk.

These first nationally representative estimates of public fungal disease awareness demonstrate major gaps, indicating a need for continued efforts to strengthen education messages, particularly for groups at higher risk and those with lower educational attainment. These data also provide a baseline for future studies to evaluate fungal disease knowledge, attitudes, and behaviors in more detail.

**Summary**

**What is already known about this topic?**

Invasive fungal diseases cause considerable morbidity and mortality. Awareness is essential for early diagnosis and treatment.

**What is added by this report?**

Public awareness of invasive fungal diseases was low in a 2019 survey of 3,624 adults; approximately two thirds of respondents had never heard of any of the diseases on the survey.

**What are the implications for public health practice?**

These results are the first estimates of nationwide public awareness of fungal diseases and serve as a baseline for future studies to assess knowledge gaps. Continued educational efforts to improve awareness are needed.

## Footnotes

* CDC licensed these data from Porter Novelli Public Services. Although Porter Novelli Public Services and its vendors are not subject to CDC IRB review, they do adhere to all professional standards and codes of conduct set forth by the Council of American Survey Research Organizations. Respondents are informed that their answers are being used for market research and they may refuse to answer any question at any time.

## References

[1] Benedict K, Jackson BR, Chiller T, Beer KD. Estimation of direct healthcare costs of fungal diseases in the United States. Clin Infect Dis 2019;68:1791–7. 10.1093/cid/ciy776 30204844 PMC6409199

[2] Vallabhaneni S, Mody RK, Walker T, Chiller T. The global burden of fungal diseases. Infect Dis Clin North Am 2016;30:1–11. 10.1016/j.idc.2015.10.004 26739604

[3] Tsang CA, Anderson SM, Imholte SB, Enhanced surveillance of coccidioidomycosis, Arizona, USA, 2007–2008. Emerg Infect Dis 2010;16:1738–44. 10.3201/eid1611.100475 21029532 PMC3294516

[4] Khan M, Brady S, Adame G, Komatsu K. Awareness and knowledge of coccidioidomycosis in Arizona: findings from the 2016 Behavioral Risk Factor Surveillance System. In: 10th International Conference on Emerging Infectious Diseases, Atlanta, Georgia, August 26–29, 2018.

[5] Benedict K, McCotter OZ, Brady S, Surveillance for coccidioidomycosis—United States, 2011–2017. MMWR Surveill Summ 2019;68(No. SS-7). 10.15585/mmwr.ss6807a1 31538631 PMC6756189

[6] Galgiani JN, Ampel NM, Blair JE, 2016 Infectious Diseases Society of America (IDSA) clinical practice guideline for the treatment of coccidioidomycosis. Clin Infect Dis 2016;63:112–46. 10.1093/cid/ciw360 27470238

[7] Benedict K, McCracken S, Signs K, Enhanced surveillance for histoplasmosis—9 states, 2018–2019. Open Forum Infect Dis 2020;7. 10.1093/ofid/ofaa343 PMC7491707 32964064

[8] Ashraf N, Kubat RC, Poplin V, Re-drawing the maps for endemic mycoses. Mycopathologia 2020. Epub February 10, 2020. 10.3201/eid2408.171595 32040709 PMC7416457

[9] Rathod SD, Buffler PA. Highly-cited estimates of the cumulative incidence and recurrence of vulvovaginal candidiasis are inadequately documented. BMC Womens Health 2014;14:43. 10.1186/1472-6874-14-43 24612727 PMC3975582

[10] CDC. Health concerns associated with mold in water-damaged homes after Hurricanes Katrina and Rita—New Orleans area, Louisiana, October 2005. MMWR Morb Mortal Wkly Rep 2006;55:41–4. 16424858

